# Strengthening the interpretation of longitudinal EQA data through the sporadic inclusion of native human sera in general clinical chemistry

**DOI:** 10.3389/fmolb.2026.1869542

**Published:** 2026-09-24

**Authors:** Laura Vierbaum, Patricia Kaiser, Ingo Schellenberg, Folker Wenzel, Mario Thevis, Manfred Falck

**Affiliations:** 1 INSTAND e.V., Society for Promoting Quality Assurance in Medical Laboratories, Duesseldorf, Germany; 2 Institute of Biochemistry/Center for Preventive Doping Research, German Sport University Cologne, Cologne, Germany; 3 Faculty of Medical and Life Sciences, Furtwangen University, Villingen Schwenningen, Germany

**Keywords:** cholesterol, commutability, creatinine, external quality assessment (EQA), sodium ion-selective electrode (ISE), trueness, urea, enzymatic assay

## Abstract

**Introduction:**

Comparative external quality assessment (EQA) surveys using native human sera alongside routinely distributed processed EQA materials (EQAMs) provide valuable information on measurement procedure (MP) trueness and the status of standardization. They also allow EQA providers to retrospectively identify critical or less critical analyte/MP pairings regarding the commutability of processed EQAMs. This study evaluates the benefit of interpreting such commutability-related indications in the context of longitudinal EQA data, for cholesterol, urea, creatinine (enzymatic), creatinine (kinetic), γ-glutamyl transferase (GGT), and sodium.

**Methods:**

In the comparative EQA, nearly 400 results were obtained for each of four native human sera and two processed EQAMs. Mixed-effects models were used to estimate analyte/MP-specific mean natural logarithm (ln) bias to reference measurement values for native and processed EQAMs, mean differences in ln bias between sample types, and based on longitudinal EQA data between 08/2023 and 10/2024, MP/batch-specific mean ln bias.

**Results:**

In the comparative EQA study, MP-specific mean differences in bias between sample types were generally small, with most meeting the analyte-specific commutability criteria. An exception among native EQAMs was observed for GGT, showing differences in bias of more than −7% and pronounced variabilities in MP-specific mean bias (standard deviation (SD) in percentage points: 3.9–10.2), likely associated with low enzyme activities. In contrast, most analyte/MP-specific bias in native EQAMs was highly consistent (SD: enzymatic assays 0.3–3.1, creatinine (kinetic) 1.7–6.6, sodium 0.21–0.42). Longitudinal data showed higher variabilities for processed EQAMs for multiple analyte/MP pairings (SD: enzymatic assays 1.0–10.0, creatinine (kinetic) 2.1–6.5, sodium 0.38–1.00), indicating higher MP susceptibilities to synthetic EQAM matrices. Substantial shifts in bias ranges between sample types were observed for cholesterol and urea across most MPs, and for individual MPs for the remaining analytes.

**Conclusion:**

In some processed EQAM batches, bias was markedly greater than observed for the batches in the comparative EQA survey, indicating that the limited number of batches in such studies do not allow generalized interpretation on commutability, and requires batch-specific assessment. However, when combined with longitudinal monitoring of MP-specific bias, a comprehensive picture emerges of MP trueness, MP susceptibility to interferences and commutability of EQAMs, thereby supporting MP and control material manufacturers in quality improvement.

## 1 Introduction

Longitudinal analyses of external quality assessment (EQA) data can provide valuable insights into the degree of standardization among different measurement procedures (MPs) (Ceriotti, 2014; Vierbaum et al., 2024b; Wojtalewicz et al., 2023). However, EQA results that reliably reflect the performance of MPs in routine clinical practice and thus, allow for reliable result evaluations for trueness - relative to reference measurement values (RMVs) - require the use of well-characterized and particularly commutable control materials (Badrick et al., 2022; Badrick et al., 2018; Delatour et al., 2019; Miller et al., 2011a; Miller et al., 2011b). However, due to frequently distributed EQA materials (EQAMs) and thus the need for a high number of batches for a broad range of analytes, it is challenging for EQA providers to comprehensively assess commutability, as such investigations are time-consuming and resource-intensive (Vierbaum et al., 2024a). Therefore, alternative approaches are needed that strike a balance between practicability and reasonable costs and time expenditure, while taking into account inevitable study limitations and, ultimately, the conclusiveness of the study outcomes.

In a previous study, we sporadically used native human sera in the EQA program for steroid hormones measurement over a 2-year period, alongside the regularly distributed processed EQAMs (Vierbaum et al., 2026). Such an approach allows for the assessment of trueness of individual MPs, relative to the RMV, thereby enabling evaluation of the status of standardization and providing insights into the measurement susceptibility to interferences of individual MPs. A key strength of this approach is the use of average measurements from several participating laboratories, whereby laboratory- and user-specific bias are largely mitigated, resulting in a representative estimate of the mean bias that reflects the analytical performance of the respective MPs in routine application. In addition to MP performance bias, comparative EQA studies proved useful for providing further insights into the suitability of processed control materials by leveraging existing EQA infrastructure, as commutability-related bias represents the other major contribution to the observed total mean bias in EQA.

Apart from individual inconclusive results due to intrinsically limited numbers of native materials in EQA studies, this framework helped distinguish between critical and less critical analyte/MP pairings with respect to commutability. Thus, it was possible to determine whether targeted, more in-depth commutability assessments–using either additional EQA surveys with commutable materials or conventional study designs–are indispensable for specific analyte-MP pairings.

The present study aims to evaluate the benefit of interpreting findings from a comparative EQA survey using native human sera, on MP performances and suitability of control materials, in the context of longitudinal EQA data derived from additional processed EQAMs. For this purpose, interlaboratory results for the measurement of cholesterol, urea, creatinine, γ-glutamyl transferase (GGT), and sodium from INSTAND–Society for Promoting Quality in Medical Laboratories e.V (ISO/IEC, 2023). – were evaluated.

## 2 Materials and methods

### 2.1 Processed EQAMs

The processed EQAMs for the measurement of cholesterol, urea, creatinine, GGT, and sodium were commercially available control materials from three different manufacturers, hereafter referred to as the anonymized manufacturers M_A_, M_B_, and M_C_. Batches were coded using the notation BX (M_z_), where BX denotes the batch identifier (X = 1–7) and M_z_ the manufacturer identifier (z = A–C). All EQAMs were pooled human sera with spiked analyte concentrations, with 5-mL aliquots of each batch subsequently lyophilized. According to the manufacturer’s specifications, the samples are stable for 3 years when stored at 2 °C–8 °C. The homogeneity and stability of the EQAMs were confirmed to be in accordance with DIN EN ISO/IEC 17043:2023 (ISO/IEC, 2023). Samples were shipped overnight to participating laboratories at ambient temperature.

### 2.2 Fresh native human sera

#### 2.2.1 Selection of donors

As the analytical performance of different MPs may depend on analyte concentration, efforts were made to obtain single-donor sera with analyte concentrations similar to those in processed EQAMs. According to the Guideline of the German Medical Association on Quality Assurance in Medical Laboratory Examinations (Rili-BÄK) (German Medical Association, 2023), control materials should cover clinically relevant concentration ranges, which includes values close to cut-off points and pathological ranges. In line with these target concentrations, the manufacturer selected ten potential donors from a pool of previously characterized individuals, whose blood parameters were screened for suitability 2 months prior to the EQA survey. The interval between pre-characterization and donation reflected the practical framework of scientific donor programs, in which predefined inter-donation intervals are required for repeat blood donations. Although analyte concentrations may change over time, the donors’ known clinical and laboratory profiles, as confirmed during screening, increased the likelihood of selecting individuals with abnormal or borderline analyte concentrations. Based on these measurements, four donors were finally selected for the preparation of fresh, native EQAMs, ensuring that at least some analytes in each donation showed elevated or reduced values compared with the reference range.

#### 2.2.2 Preparation of native EQAMs

Four sera were obtained from standard whole blood donations by clotting and subsequent centrifugation, according to the supplier’s standard operating procedures. No substances were added to the sera for the purpose of manipulation or stabilization. Sera were tested to be negative for human immunodeficiency virus, hepatitis B virus and hepatitis C virus. From each serum, a bulk volume of 15 mL was retained for stability testing and RMV assignment in the calibration laboratory at INSTAND. Aliquots of 0.4 mL were prepared, resulting in 493 aliquots per serum, limited by the lowest available serum volume. Each serum batch was assigned an anonymous EQAM batch identifier (N1–N4). Both the bulk volume and the aliquots were delivered to INSTAND within 28 h after sampling. Sample preparation, shipment, and handling at INSTAND were performed at 2 °C–8 °C.

#### 2.2.3 Stability testing

Stability testing of cholesterol, creatinine, urea and GGT in the native sera was performed using Beckman-Coulter AU480 (for urea measurements DiaSys, all others Beckman reagents). Creatinine was measured by both an enzymatic assay and the Jaffé kinetic method. The initial measurement was performed upon sample receipt at INSTAND, i.e., one day after blood collection. Follow-up measurements were conducted on days three, four, and seven after collection. Native EQAMs were stored at 2 °C–8 °C, reflecting the conditions during the EQA survey.

### 2.3 Routine EQA procedure

The INSTAND EQA scheme for the measurement of cholesterol, urea, creatinine, GGT, and sodium is routinely conducted worldwide six times a year. In routine EQA survey, two processed EQAMs with different levels are to be tested by the participating laboratories (see Section 2.1). EQAMs are shipped to the laboratories at ambient temperature and must be analyzed within two and a half weeks. Participants should store the materials at 2 °C–8 °C until analysis. The quantitative results for cholesterol, urea, creatinine, GGT, and sodium can be reported by the participating laboratories via the platform ‘RV-Online’ (http://rv-online.instandev.de). In addition to the measurement results, participants should inform INSTAND as to which device, method and reagent they used.

In EQA evaluations, laboratories are allowed maximum deviations from the respective RMV of ±13% for cholesterol, ±20% for urea, ±20% for creatinine, ±21% for GGT, and ±5% for sodium to receive certification, as stipulated by the Rili-BÄK guideline (German Medical Association, 2023).

### 2.4 EQA using fresh native sera

In the EQA survey conducted in 05/2024, four native EQAMs (batch identifiers N1-N4) were distributed to 493 participating laboratories in Germany in addition to the routinely provided processed EQAMs (batch identifiers B1 (M_A_), B2 (M_A_)). To supplement relatively small collectives (excluding RO), 25 additional laboratories from ten other European Union countries with typically rapid turnaround times were individually selected and considered for the distribution of the EQAMs. As the native EQAMs contained no stabilizing additives, they were shipped within 50 h of sample donation to participating laboratories, with cooling pads included in the package without direct contact with the samples. Quantitative results for the analytes, along with the measurement time points, as well as the instrument, method, and assay used, were submitted via the RV-Online platform.

The stability of the measurement of cholesterol, urea, creatinine, GGT, and sodium in the native sera was monitored throughout the EQA survey (see Section 2.2).

### 2.5 Reference measurement procedures

For all EQAMs, the RMVs of cholesterol, urea, creatinine, GGT, and sodium were assigned by the INSTAND calibration laboratory, accredited according to DIN EN ISO/IEC 17025:2018 and DIN EN ISO/IEC 15195:2019 (ISO/IEC, 2018a; ISO/IEC, 2018b).

Metrological traceability was established using primary reference materials (cholesterol NIST SRM 911c, urea NIST SRM 912b, creatinine NIST SRM 914a, NaCl NIST 919b). Six measurements were performed for each target value - two measurements per day over three consecutive days. Measurement uncertainty was estimated to each target value on the basis of a measurement uncertainty budget.

Isotope dilution gas chromatography/mass spectrometry (GC-ID/MS) was used as the established reference measurement procedure (RMP) for total cholesterol, urea, and creatinine in serum (Kessler and Siekmann, 1999; Stöckl and Reinauer, 1993; Thienpont et al., 1993). For cholesterol value assignment, the samples were gravimetrically spiked with ^13^C_2_-cholesterol used as internal standard, equilibrated, hydrolyzed with fresh methanolic KOH and extracted with n-hexane. Derivatization was performed with *N*-Methyl-*N*-(trimethylsilyl)trifluoroacetamide (MSTFA). Gas chromatography/mass spectrometry (GC-MS) measurements were done at the mass-to-charge ratios of *m/z* 458 and *m/z* 460. For urea value assignment, the samples were gravimetrically spiked with ^13^C_1_,^15^N_2_-urea used as internal standard, equilibrated and the proteins were then precipitated with ethanol, centrifuged, and the supernatant was evaporated to dryness. Derivatization was performed with Damdee solution in pyridine. GC-MS measurements were done at the mass-to-charge ratios of *m/z* 171 and *m/z* 174. The creatinine target value assignments were performed by gravimetrically spiking the materials with ^13^C_1_-creatinine used as internal standard. The samples were then equilibrated and ultrafiltrated before the creatinine was separated by high pressure liquid chromatography (HPLC) using a C18 column. Derivatization was performed using N-Methyl-N-tert-butyldimethylsilyltrifluoroacetamide (MBDSTFA) in pyridine. GC-MS measurements were performed at *m/z* 298 and *m/z* 299.

For the determination of RMVs for sodium inductively coupled plasma/mass spectrometry (ICP-MS) was used. The samples were gravimetrically spiked with aluminum used as internal standard and diluted approximately 10,000 times with 4 mM nitric acid. ICP-MS measurements were performed at *m/z* 22.990 and *m/z* 26.982 (Kramer et al., 2013).

Target values for GGT were assigned according to The International Federation of Clinical Chemistry and Laboratory Medicine (IFCC) RMP as described in (Schumann et al., 2002).

### 2.6 Data analysis and statistics

The aim of the analyses was first to compare EQA data for cholesterol, urea, creatinine, GGT, and sodium between native and processed EQAMs of the survey in 05/2024, analogous to the previously proposed approach by Vierbaum et al. (Vierbaum et al., 2026), and second to evaluate cross-survey batch variability for the two sample types by involving longitudinal EQA data collected between 08/2023 and 10/2024.

Creatinine results obtained using enzymatic assays or Jaffé kinetic methods were considered separately, due to their differing analytical specificity and limited cross-method comparability (Boss et al., 2023; Ricós et al., 2019; Thomas, 2025). Seven main manufacturer collectives formed the basis for all subsequent mixed-effects model analyses: Abbott (ARCHITECT/Alinity: AB), Beckman Coulter (UniCel DxC: BE; Olympus AU: OL), Siemens Healthineers (Atellica CH: SI), Becton Dickinson (ADVIA 1800/2400: BG), Siemens Diagnostics (Dimension/Dimension Vista: BW), and Roche (INTEGRA/cobas c: RO) (Supplementary Table S1).

For data preparation, EQA results were natural logarithmic (ln) transformed and normalized to the respective RMV, hereinafter referred to as ‘ln bias’. Outlier exclusion was performed based on the ‘ln bias’ for each pairing of analyte, manufacturer collective, sample type and survey using a Grubbs’ test (α = 0.01). Subsequently, enzymatic measurement results of creatinine using the BE reagent were excluded from the comparative analysis of the EQA data in 05/2024 because fewer than three observations remained after outlier exclusion. Furthermore, all creatinine (kinetic) results for EQAM batch B1 (M_A_) were excluded from the comparative as well as longitudinal analysis. During routine EQA evaluation, this batch exhibited exceptionally large deviating values from the RMV across all collectives clearly indicating a material-derived issue that rendered it unsuitable for laboratory performance assessment. Consequently, the batch was rejected for routine EQA use and discarded prior to this study. Its exclusion from the present analyses did not affect the overall interpretation of the study. And rendered the material unsuitable for routine EQA use. Exclusion of this batch did not affect the overall interpretation of the study.

#### 2.6.1 EQA study with native and processed materials

For the comparative EQA analysis (Vierbaum et al., 2026), fixed effects included ‘sample type’ (native, processed) and ‘manufacturer collective’ (where applicable: AB, BE, OL, RO, SI, BG, and BW), as well as the interaction ‘sample type*manufacturer collective’. Random effects comprised ‘batch identification number’ nested within ‘sample type’, and ‘participant identification number’ nested within ‘manufacturer collective’. In response, mean ln bias of the two sample types and corresponding mean differences in ln bias (native minus processed EQAMs) were estimated with 95%-confidence intervals (CI), and plotted for each manufacturer collective (panels A and B of Figures 1–6). The figures were grouped by analytes, cholesterol (Figure 1), urea (Figure 2), creatinine (enzymatic) (Figure 3), creatinine (kinetic) (Figure 4), GGT (Figure 5), and sodium (Figure 6). In order to make the ln-transformed bias easier to interpret, the y-axis was scaled in percentages. The relationship is as follows:

$$bias\;\left[\%\right]=\left({e\;}^{\ln\;bias}-1\right)\;x\;100$$

**FIGURE 1:**
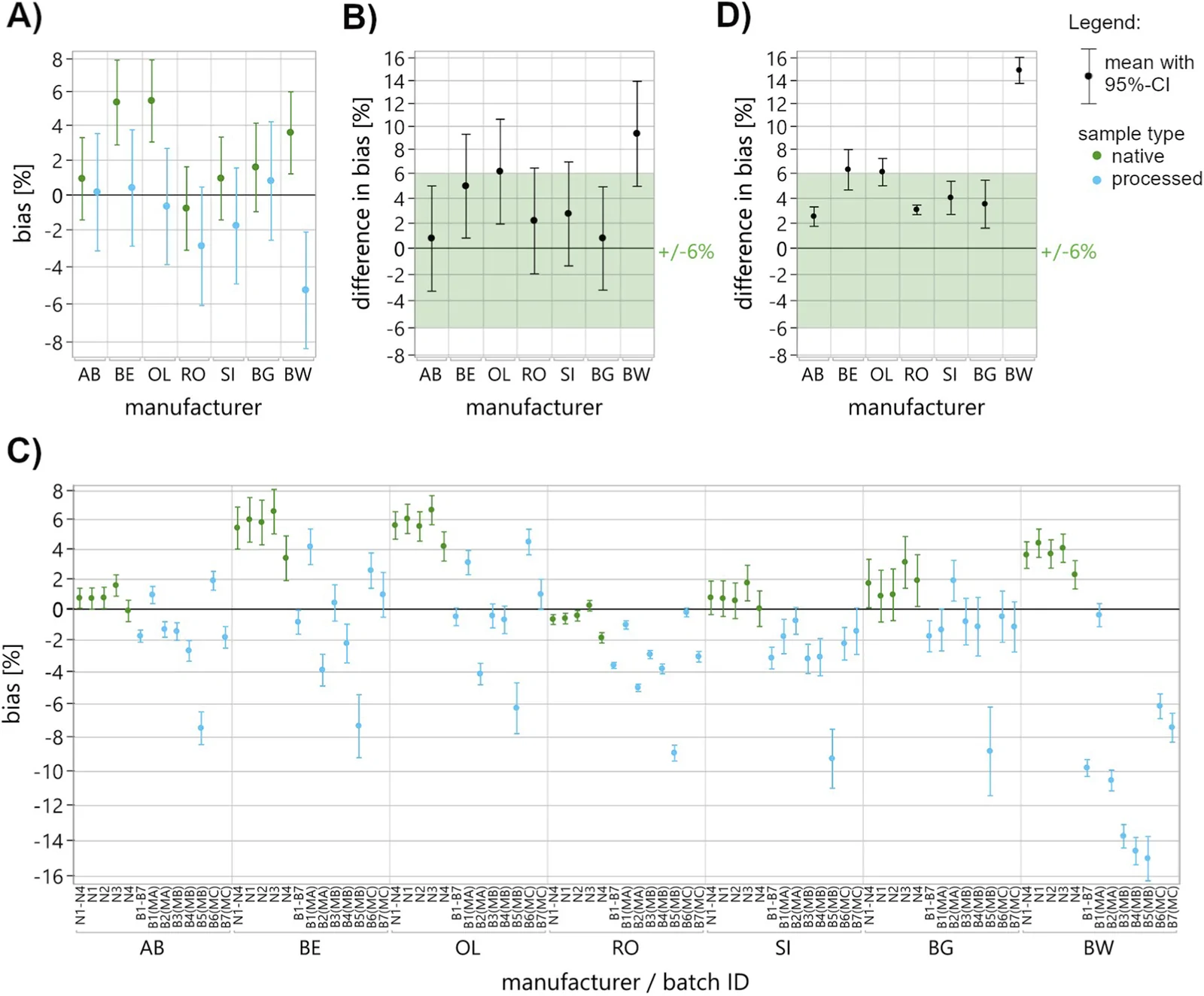
For cholesterol, mixed-effects model estimates of measurement procedure (MP)-specific mean ln bias for native (green) and processed (blue) EQA materials, and of MP-specific mean differences in bias between the sample types (native minus processed EQAMs). **(A,B)** Results from the comparative EQA study in 05/2024 (month/year) using four native and two processed EQAMs; **(C,D)** results from longitudinal EQA data from eight surveys between 08/2023 and 10/2024, including five additional processed EQAM batches. All estimates are shown as mean value with 95%-confidence interval (CI), for each MP collective (manufacturers AB, BE, OL, RO, SI, BG, BW), using a percentage-scaled y-axis. The green areas highlight the maximum allowable mean difference in bias of ±6% between the two sample types to assess the processed EQAM batches as commutable.

**FIGURE 2:**
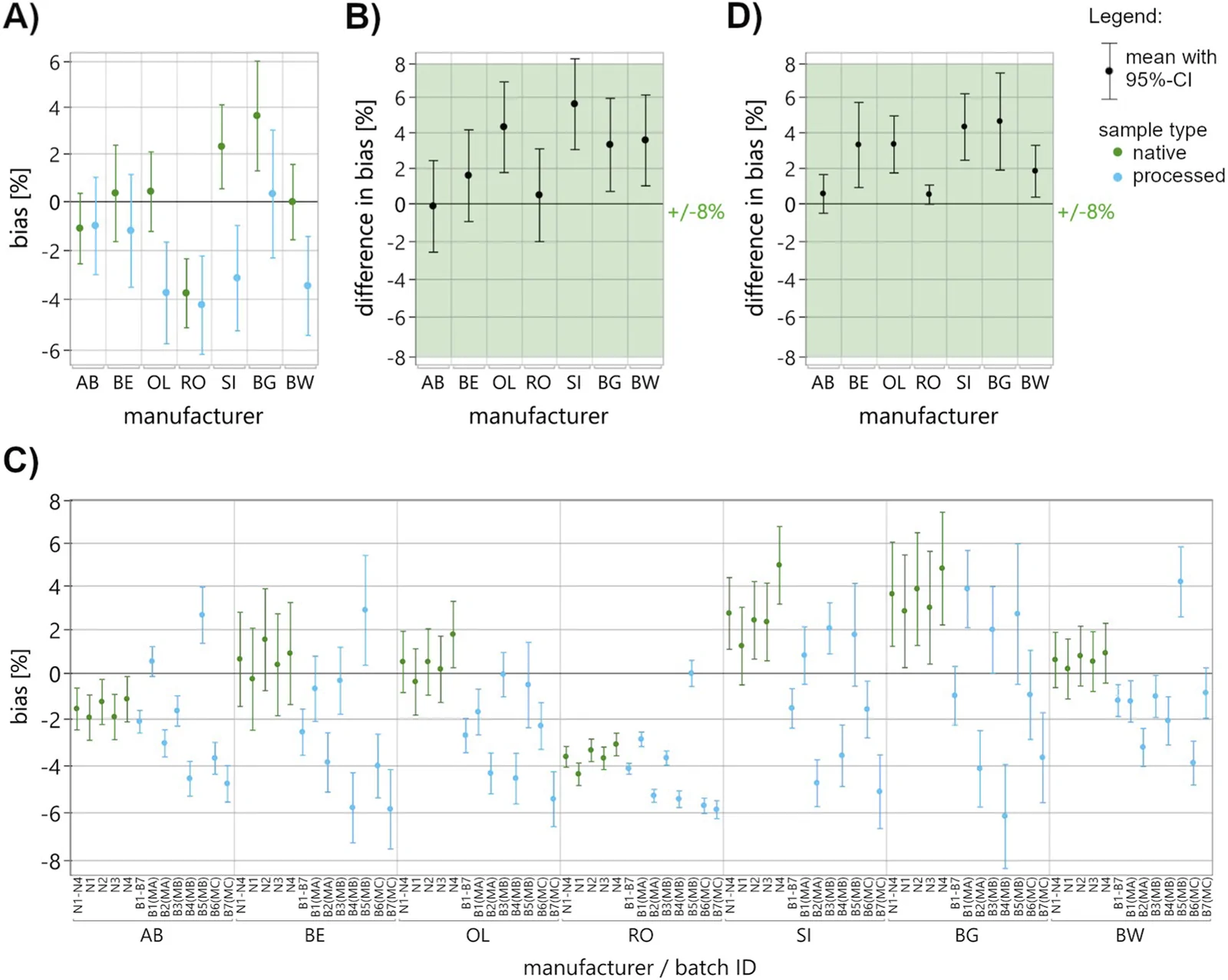
For urea, mixed-effects model estimates of measurement procedure (MP)-specific mean ln bias for native (green) and processed (blue) EQA materials (EQAMs), and of MP-specific mean differences in bias between the sample types (native minus processed EQAMs). **(A,B)** Results from the comparative EQA study in 05/2024 (month/year) using four native and two processed EQAMs; **(C,D)** results from longitudinal EQA data from eight surveys between 08/2023 and 10/2024, including five additional processed EQAM batches. All estimates are shown as mean value with 95%-confidence interval (CI), for each MP collective (manufacturers AB, BE, OL, RO, SI, BG, BW), using a percentage-scaled y-axis. The green areas highlight the maximum allowable mean difference in bias of ±8% between the two sample types to assess the processed EQAM batches as commutable.

**FIGURE 3:**
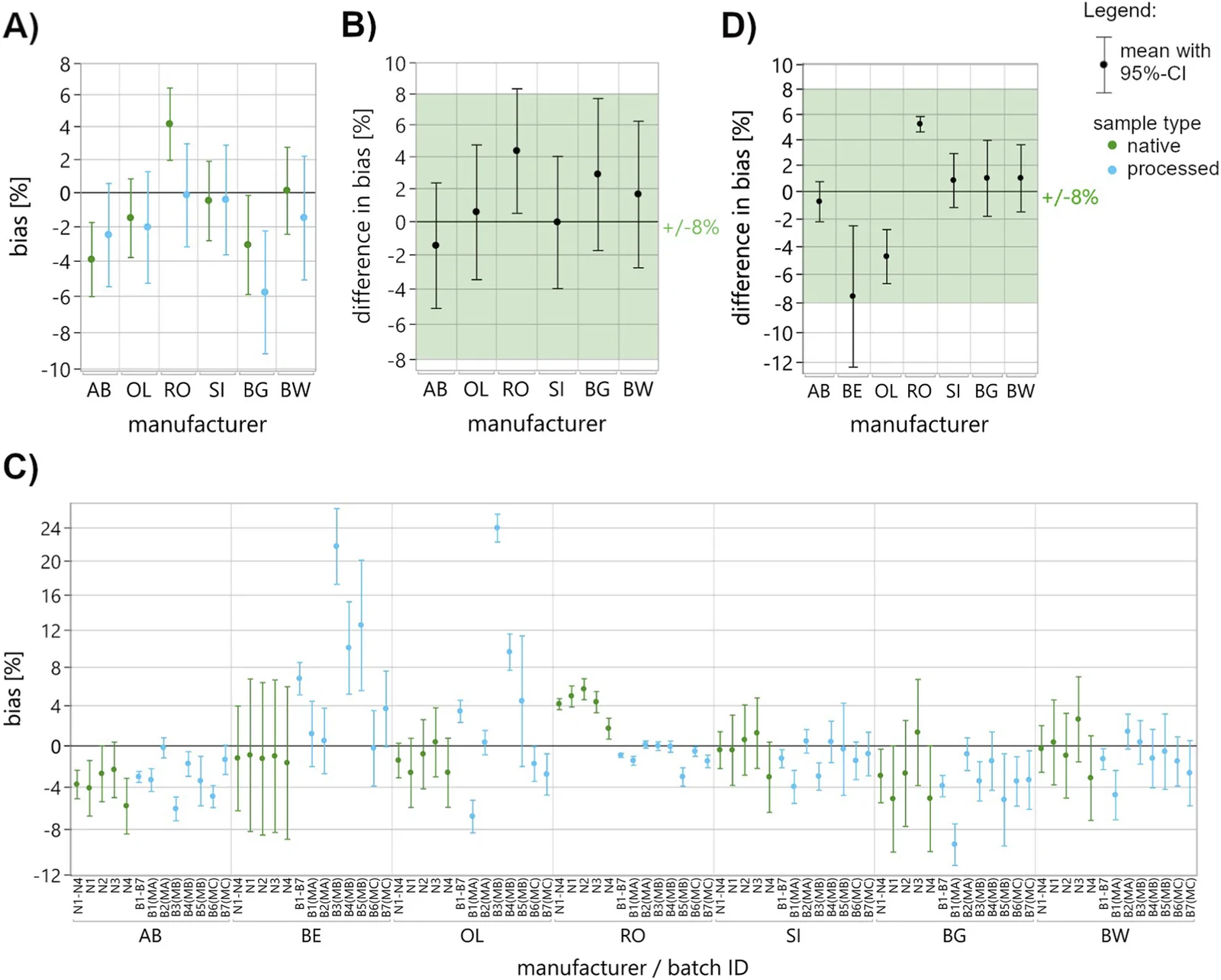
For creatinine (enzymatic), mixed-effects model estimates of measurement procedure (MP)-specific mean ln bias for native (green) and processed (blue) EQA materials (EQAMs), and of MP-specific mean differences in bias between the sample types (native minus processed EQAMs). **(A,B)** Results from the comparative EQA study in 05/2024 (month/year) using four native and two processed EQAMs; **(C,D)** results from longitudinal EQA data from eight surveys between 08/2023 and 10/2024, including five additional processed EQAM batches. All estimates are shown as mean value with 95%-confidence interval (CI), for each MP collective (manufacturers AB, BE, OL, RO, SI, BG, BW), using a percentage-scaled y-axis. The BE collective is not included in the comparative EQA study due to a lack of data. The green areas highlight the maximum allowable mean difference in bias of ±8% between the two sample types to assess the processed EQAM batches as commutable.

**FIGURE 4:**
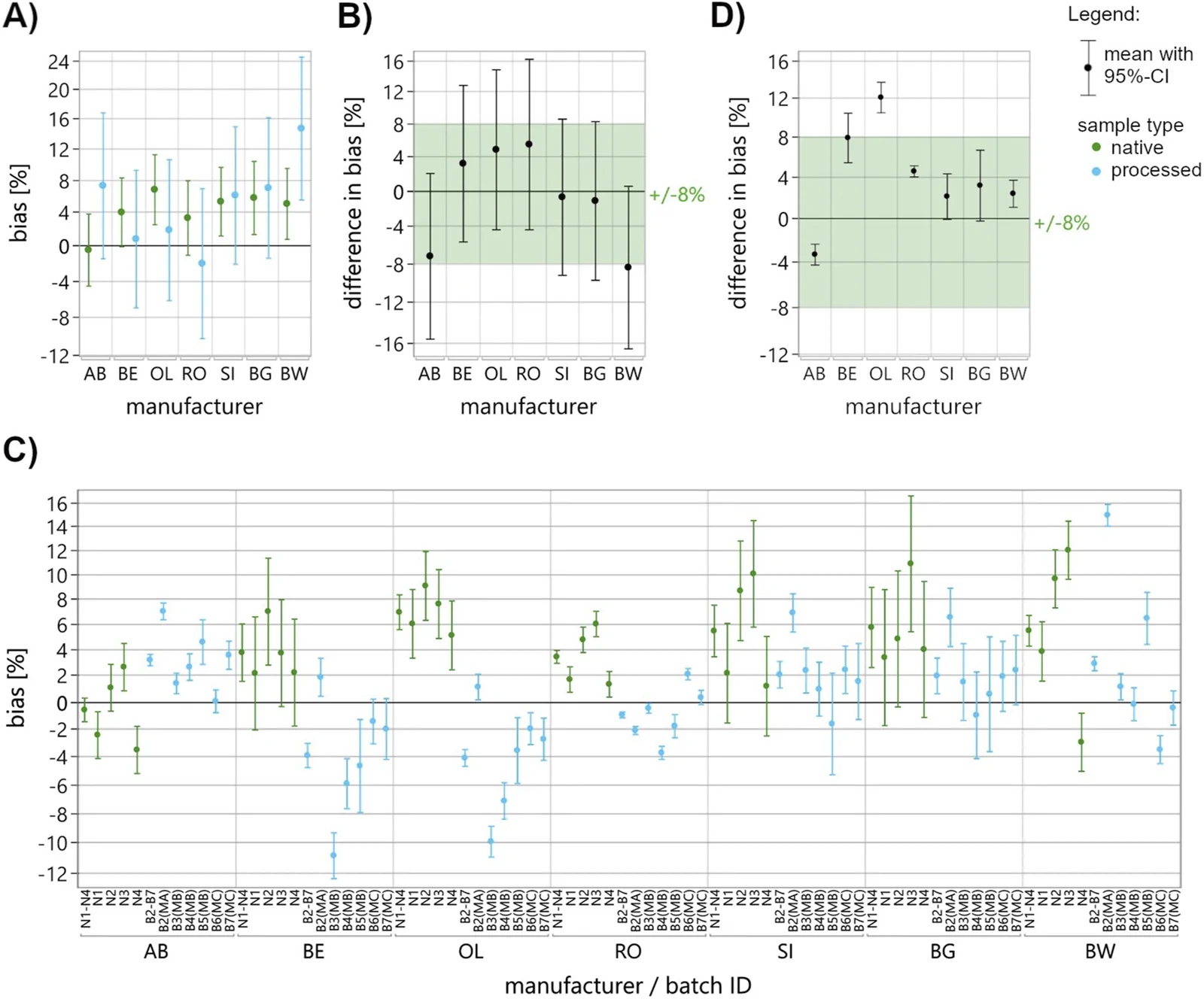
For creatinine (kinetic), mixed-effects model estimates of measurement procedure (MP)-specific mean ln bias for native (green) and processed (blue) EQA materials (EQAMs), and of MP-specific mean differences in bias between the sample types (native minus processed EQAMs). **(A,B)** Results from the comparative EQA study in 05/2024 (month/year) using four native and one processed EQAMs; **(C,D)** results from longitudinal EQA data from eight surveys between 08/2023 and 10/2024, including five additional processed EQAM batches. Data for EQAM batch B1 (MA) were excluded from the analyses due to exceptionally large deviating bias across all collectives. All estimates are shown as mean value with 95%-confidence interval (CI), for each MP collective (manufacturers AB, BE, OL, RO, SI, BG, BW), using a percentage-scaled y-axis. The green areas highlight the maximum allowable mean difference in bias of ±8% between the two sample types to assess the processed EQAM batches as commutable.

**FIGURE 5:**
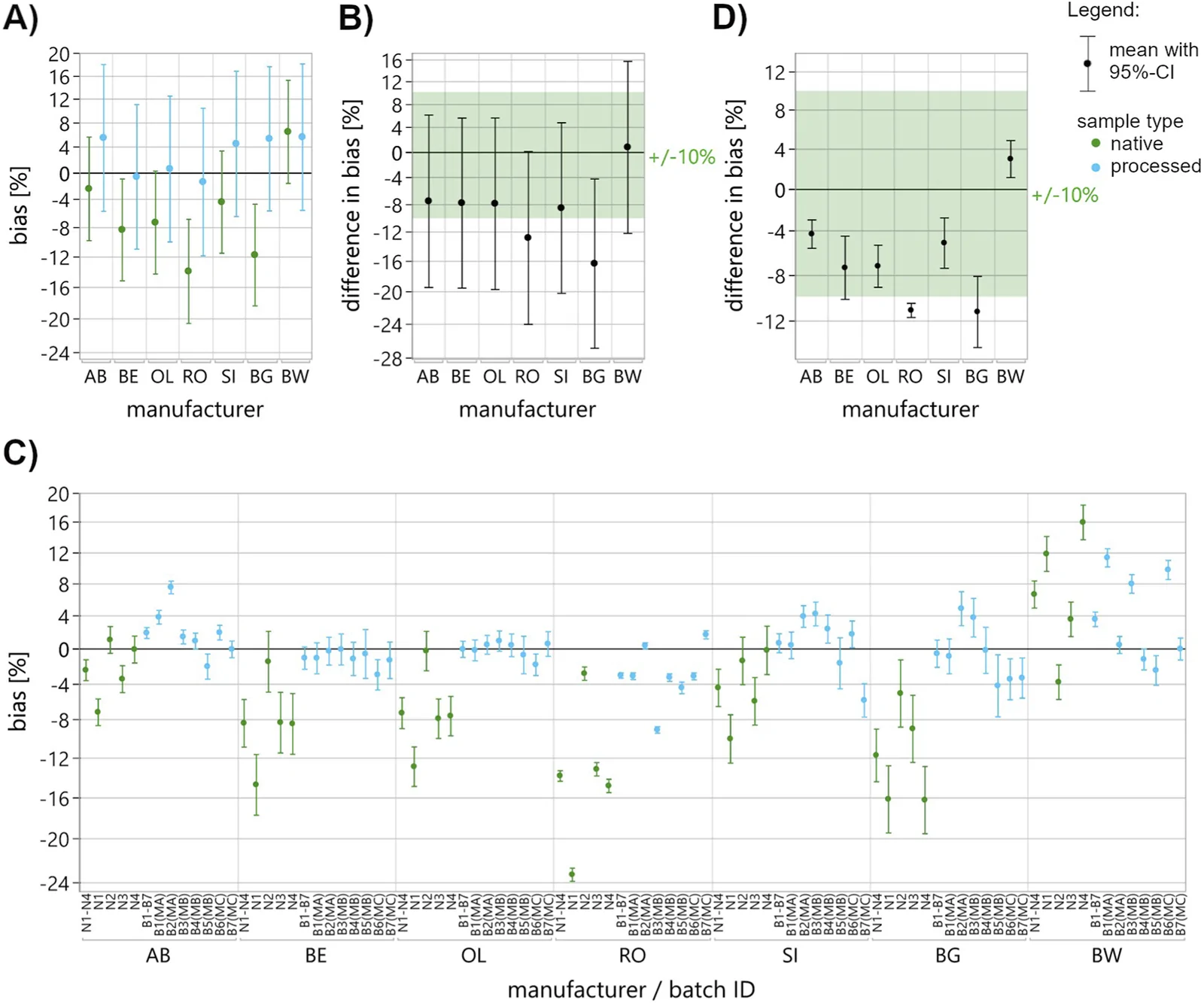
For γ-glutamyl transferase (GGT), mixed-effects model estimates of measurement procedure (MP)-specific mean ln bias for native (green) and processed (blue) EQA materials (EQAMs), and of MP-specific mean differences in bias between the sample types (native minus processed EQAMs). **(A,B)** Results from the comparative EQA study in 05/2024 (month/year) using four native and two processed EQAMs; **(C,D)** results from longitudinal EQA data from eight surveys between 08/2023 and 10/2024, including five additional processed EQAM batches. All estimates are shown as mean value with 95%-confidence interval (CI), for each MP collective (manufacturers AB, BE, OL, RO, SI, BG, BW), using a percentage-scaled y-axis. The green areas highlight the maximum allowable mean difference in bias of ±10% between the two sample types to assess the processed EQAM batches as commutable.

**FIGURE 6:**
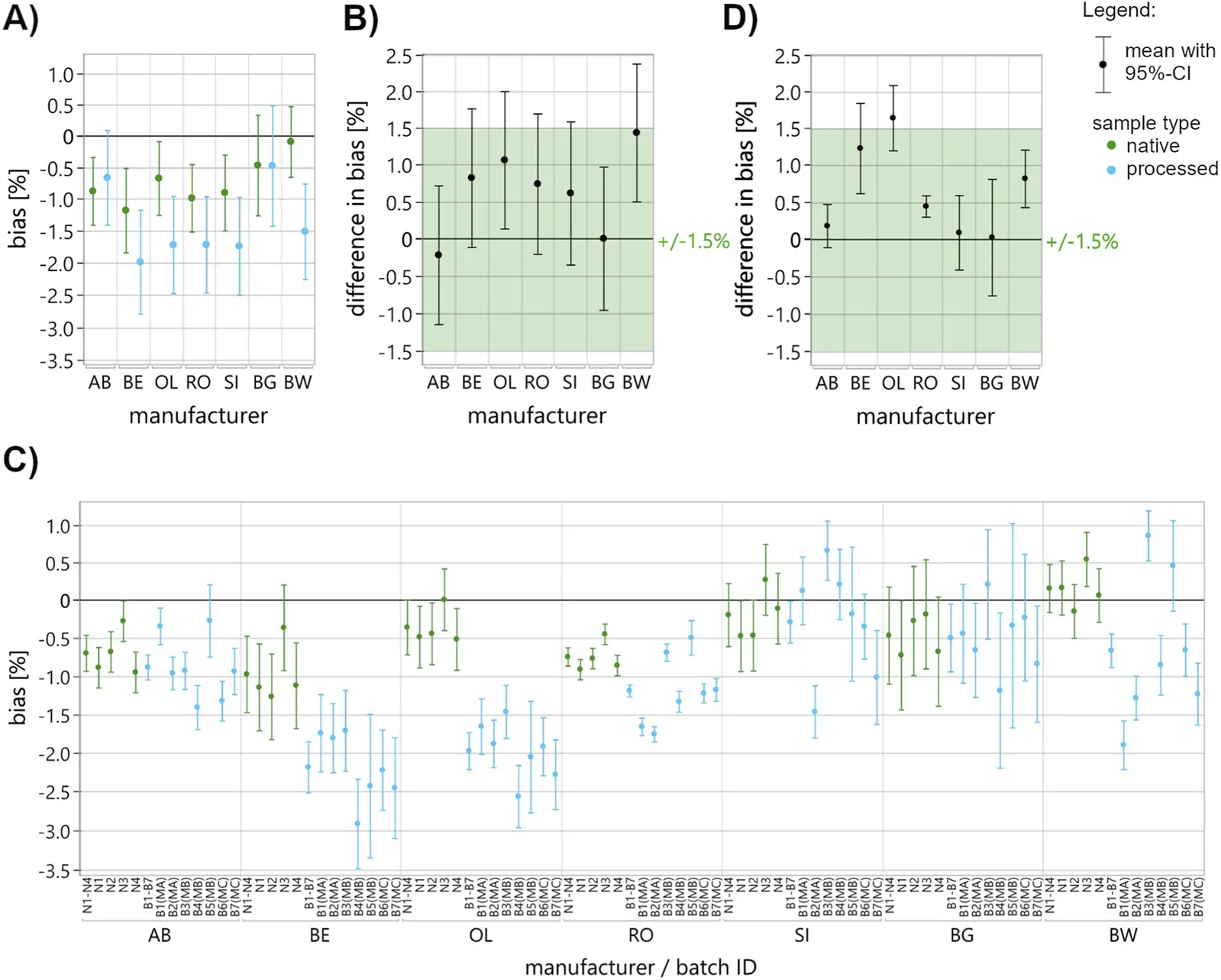
For sodium, mixed-effects model estimates of measurement procedure (MP)-specific mean ln bias for native (green) and processed (blue) EQA materials (EQAMs), and of MP-specific mean differences in bias between the sample types (native minus processed EQAMs). **(A,B)** Results from the comparative EQA study in 05/2024 (month/year) using four native and two processed EQAMs; **(C,D)** results from longitudinal EQA data from eight surveys between 08/2023 and 10/2024, including five additional processed EQAM batches. All estimates are shown as mean value with 95%-confidence interval (CI), for each MP collective (manufacturers AB, BE, OL, RO, SI, BG, BW), using a percentage-scaled y-axis. The green areas highlight the maximum allowable mean difference in bias of ±1.5% between the two sample types to assess the processed EQAM batches as commutable.

Due to the absence of universally standardized commutability limits, study-specific criteria were established using a pragmatic error-budget approach. The total allowable deviation defined by the EQA acceptance criteria (German Medical Association, 2023) was interpreted as comprising both a potential non-commutability-related and an MP-related contribution. Accordingly, for the assessment of EQAMs, a maximum of 40%–50% of the EQA criterion was predefined as the study-specific allocation for the allowable non-commutability bias. The remaining proportion represented the effective analytical performance margin for the evaluated MPs within the EQA survey after accounting for potential non-commutability effects and was cross-checked against analytical performance specifications (Aarsand et al., 2026; Ricós et al., 1999; Thomas, 2025) as an external plausibility benchmark. The predefined commutability limits were refined in an analyte-specific manner where clinically justified, applying stricter criteria for analytes with higher clinical sensitivity, particularly those with narrow reference intervals (e.g., urea, creatinine, sodium). Final values were rounded to integer percentages, except for sodium, where one decimal place was retained due to the smaller magnitude of its scale. Accordingly, commutability criteria were set at ±6% for cholesterol, ±8% for urea, ±8% for creatinine, ±10% for GGT and ±1.5% for sodium. To assess the observed differences in bias, the analyte-specific acceptance criteria ranges were highlighted in green (panel B and D of Figures 1–6).

#### 2.6.2 Analysis of longitudinal EQA data

In order to evaluate cross-survey variability in batch-specific mean ln bias, a second mixed-effects model was conducted based on longitudinal EQA data between 08/2023 and 10/2024. During this period, a total of seven different processed EQAM batches (B1-B7), assigned to manufacturer M_A_-M_C_, were used with varying frequency, up to four times within the period examined. As fixed effects the ‘manufacturer collective’, the ‘batch identification number’ and their interaction ‘manufacturer collective*batch identification number’ were defined. The ‘participant identification number’ nested in the ‘manufacturer collective’ was set as random effect. Given that the sample type-specific data were primarily independent over the longitudinal period - with partially paired data available only for the survey in 05/2024 - the data from the native and processed EQAM batches were analyzed separately. For each analyte and manufacturer collective, the batch-specific and the cross-batch mean ln bias with 95%-CI were estimated. Although derived from separate model calculations, the outcomes for both sample types were displayed in a shared plot (panel C of Figures 1–6). Again, the y-axis was scaled in percentages for easier interpretation of the illustrated data (see Section 2.5.1). Cross-batch variability in the MP-specific mean ln bias was described by the standard deviation (SD) and the range between minimum (Min.) and maximum (Max.) (Table 1). To facilitate interpretation, results were retransformed and expressed as approximate percentage differences, whereas SDs are reported in percentage points.

**TABLE 1:** Variabilities in measurement procedure (MP)-specific mean ln bias for four native and seven processed EQA materials (EQAMs), calculated based on longitudinal EQA data of eight surveys from 08/2023 to 10/2024 (month/year), for (A) the enzymatic measurements of cholesterol, urea, and creatinine (enzymatic), and (B) the measurements of creatinine (kinetic), γ-glutamyl transferase (GGT), and sodium. Because EQAM batch B5 (M_B_) exhibited pronounced mean bias for cholesterol across all MPs (manufacturers AB, BE, OL, RO SI, BG, BW) and was considered non-commutable, it was excluded from the detailed analysis of bias variability. All values were calculated as ln bias, then retransformed and expressed as approximate percentage differences; standard deviations (SDs) are reported in percentage points. The minimum (Min.) and maximum (Max.) mean bias are marked in red if mean bias of only one or none processed EQAM batch falls within the bias range of native EQAMs, indicating either a conspicuous bias shift between sample types or elevated variability in bias of processed compared to native EQAMs. The Max.-Min. ranges and the SD of the individual MPs are highlighted in red when the difference between the two sample types exceeds a factor of three, and in light red when it exceeds a factor of two.

MP	Sample type	Cholesterol (without B5 (MB)				Urea				Creatinine (enzymatic)			
		Min	Max	Max.-Min	SD	Min	Max	Max.-Min	SD	Min	Max	Max.-Min	SD
A)													
AB	native	−0.09	1.57	1.66	0.68	−1.93	−1.12	0.81	0.44	−5.81	−2.38	3.44	1.64
	processed	−2.67	1.88	4.56	1.77	−4.78	2.65	7.43	2.86	−6.07	−0.23	5.84	2.14
BE	native	3.39	6.59	3.20	1.36	−0.24	1.53	1.77	0.75	−1.72	−0.97	0.75	0.34
	processed	−3.89	4.17	8.05	3.02	−5.85	2.87	8.73	3.36	−0.28	21.71	21.98	7.71
OL	native	4.19	6.69	2.50	1.01	−0.36	1.77	2.12	0.90	−2.66	0.32	2.98	1.49
	processed	−4.13	4.49	8.63	3.09	−5.43	−0.03	5.40	2.19	−6.78	23.93	30.71	10.00
RO	native	−1.84	0.25	2.08	0.88	−4.37	−3.10	1.28	0.58	1.68	5.70	4.02	1.71
	processed	−4.99	−0.20	4.79	1.84	−5.87	0.01	5.89	2.23	−3.06	0.10	3.15	1.17
SI	native	0.05	1.73	1.68	0.70	1.24	4.95	3.71	1.53	−3.08	1.22	4.30	1.93
	processed	−3.18	−0.74	2.44	0.98	−5.12	2.04	7.16	3.17	−4.01	0.42	4.43	1.73
BG	native	0.87	3.12	2.25	1.03	2.82	4.80	1.97	0.88	−5.15	1.29	6.44	3.14
	processed	−1.33	1.89	3.22	1.21	−6.15	3.85	10.00	3.98	−9.33	−0.85	8.48	2.99
BW	native	2.29	4.41	2.12	0.91	0.22	0.92	0.71	0.31	−3.17	2.61	5.78	2.46
	processed	−14.59	−0.37	14.22	5.94	−3.90	4.18	8.08	2.65	−4.78	1.38	6.17	2.08

MP	Sample type	Creatinine (kinetic)				GGT				Sodium			
		Min	Max	Max.-Min	SD	Min	Max	Max.-Min	SD	Min	Max	Max.-Min	SD
B)													
AB	native	−3.52	2.65	6.17	2.96	−7.16	1.06	8.23	3.91	−0.947	−0.273	0.67	0.306
	processed	0.06	7.01	6.96	2.39	−2.05	7.50	9.54	3.00	−1.404	−0.268	1.14	0.442
BE	native	2.17	7.00	4.83	2.19	−14.74	−1.48	13.26	6.08	−1.264	−0.360	0.90	0.416
	processed	−10.86	1.86	12.72	4.71	−2.99	−0.06	2.93	0.99	−2.909	−1.707	1.20	0.463
OL	native	5.11	9.07	3.96	1.65	−12.91	−0.24	12.67	5.73	−0.512	0.010	0.52	0.246
	processed	−9.91	1.13	11.03	4.20	−1.84	0.93	2.77	0.97	−2.558	−1.460	1.10	0.378
RO	native	1.33	6.01	4.67	2.24	−23.29	−2.85	20.43	10.16	−0.908	−0.445	0.46	0.209
	processed	−3.73	2.10	5.83	2.09	−9.07	1.68	10.74	3.67	−1.752	−0.492	1.26	0.471
SI	native	1.19	10.06	8.87	4.35	−10.03	−0.17	9.86	4.87	−0.469	0.274	0.74	0.354
	processed	−1.63	6.90	8.52	2.74	−5.86	4.20	10.06	3.63	−1.461	0.660	2.12	0.741
BG	native	3.38	10.89	7.51	3.28	−16.26	−5.09	11.16	6.43	−0.721	−0.182	0.54	0.277
	processed	−0.99	6.54	7.53	2.48	−4.24	4.85	9.08	3.66	−1.184	0.213	1.40	0.454
BW	native	−2.96	12.02	14.98	6.62	−3.82	16.01	19.84	8.70	−0.144	0.545	0.69	0.288
	processed	−3.50	14.99	18.49	6.52	−2.49	11.30	13.79	5.67	−1.895	0.859	2.75	0.996

In addition, for repeatedly distributed processed batches across different surveys, variations in MP-specific median ln bias (Max.-Min.) were evaluated based on the longitudinal EQA data from 08/2023 to 10/2024 (Supplementary Table S2).

Based on the sample type-specific mean ln bias across all native and processed EQAM batches, respectively, MP-specific mean differences in ln bias between sample types were calculated (panel D of Figures 1–6). The corresponding confidence intervals were calculated under the assumption of independence, resulting in conservative estimates.

All statistical analyses were performed by JMP 19.0.1 from SAS Institute (Cary, North Carolina, USA).

### 2.7 Generation of images

The overlay images were generated using the Gnu image manipulator software 2.10.38.

## 3 Results

### 3.1 Analyte concentrations of native EQA materials

The preliminary screening of four donors enabled the selection of individuals covering low and high concentrations for cholesterol, urea, creatinine, and GGT in at least one serum donation. RMP assignment of the native EQAMs yielded concentrations of 133.6–206.6 mg/dL for cholesterol, 29.36–46.03 mg/dL for urea, 0.724–0.860 mg/dL and 1.122 mg/dL for creatinine, 14.5–28.0 U/L and 150.0 U/L for GGT, and 140.5–143.5 mmol/L for sodium. It should be noted that RMP-based GGT measurements of the two native EQAM batches N1 and N4 yielded values below the accredited measuring interval of 20–300 U/L. Nevertheless, the measurement uncertainty (2.5%–2.8%) was not increased compared with that associated with target value assignment within the accredited measurement interval, with 2.5% corresponding to the best measurement capability of the accredited RMP.

Except for GGT, no native EQAMs with markedly elevated pathological values were finally distributed to participating laboratories, considering an approximately reference range of >40 U/L in females and >60 U/L in males (Thomas, 2025). However, two values for cholesterol and one value each for urea and creatinine were around the upper limits of the respective reference ranges - approximately 115–200 mg/dL cholesterol, 17–43 mg/dL urea, and 0.4–1.0 mg/dL creatinine in females or 0.6–1.3 mg/dL creatinine in males (Thomas, 2025). All sodium levels were within the normal range of 135–145 mmol/L (Thomas, 2025).

### 3.2 Comparative EQA survey

In the comparative EQA survey conducted in 05/2024, approximately 80% of the laboratories that analyzed the processed EQAMs also participated in the voluntary measurement of native EQAMs. Thus, for each of the four native EQAMs, a total of 376–395 results were obtained per analyte, meaning more than 1,500 results per analyte. Only a few Grubbs outliers were excluded, with a maximum of N = 37 for urea measurement in native EQAMs; all other analytes and sample types showed ≤18 outliers (see Supplementary Table S1). Analyses were performed for each analyte/MP pairing, resulting in sample type–specific collective sizes of typically ≥30 data points for cholesterol, urea, GGT and sodium. Exceptions were observed for the BE and the BG collectives for the processed EQAMs with at least16 data. As creatinine data were stratified by enzymatic and Jaffé kinetic methods, the collective sizes were generally smaller, with two comprising fewer than 10 data points (N = 9 for enzymatic BW and N = 6 for kinetic BG).

Mixed-effects model analysis based on the EQA data in 05/2024 provided MP collective-specific bias to the RMVs for native and processed EQAMs (panel A of Figures 1–6) and MP-specific differences in bias between the sample types (panel B of Figures 1–6).

For three analytes, urea, creatinine (enzymatic), and sodium, a tendency toward negative MP-specific bias was observed for both sample types (panel A of Figures 1–3). However, slight positive bias of up to approximately 4% were noted for three analyte/MP pairings. Negative bias ranged down to −4% for cholesterol, −6% for enzymatic creatinine, and −2% for sodium measurement. In contrast, the kinetic creatinine measurement showed a clear tendency toward positive MP-specific bias for both sample types, with a maximum of approximately 15% observed for processed EQAMs using the BW reagent (Figure 4A). However, most bias remained under 8% for Jaffé kinetic creatinine measurement. For the measurement of cholesterol and GGT both positive and negative bias were estimated, ranging between −6% and 6% for cholesterol and −14% and 7% for GGT (panel A of Figures 1, 5).

Considerable discrepancies in mean ln bias between native and processed EQAMs (panel A of Figures 1–6) were reflected in substantial estimated mean differences (panel B of Figures 1–6). With the exception of GGT and some MP-specific measurements of creatinine (kinetic), a consistent trend toward higher bias in native compared to processed EQAMs was observed across analyte/MP pairings (panel A of Figures 1–6), resulting in predominantly positive mean differences in ln bias (native minus processed EQAMs) (panel B of Figures 1–6). The smallest relative differences were observed for sodium measurement with a maximum difference of 1.44% for the BW reagent, followed by 4.3% for creatinine (enzymatic)/RO and 5.6% for urea/SI (panel B of Figures 2, 3, 6). Differences in bias for cholesterol and creatinine (kinetic) were generally in a similar range, with the exceptions of 9.3% for cholesterol/BW and −7.2% and −8.4% for creatinine (kinetic)/AB and BW, respectively (panel A of Figures 1, 4). The most pronounced differences were observed for GGT, reaching −16.3% for the BG and −12.8% for the RO collective.

These differences in bias between native and processed EQAMs were largely within predefined analyte-specific commutability criteria. Only a few analyte/reagent pairings showed mean differences clearly exceeding the respective limits: primarily, 9.3% for cholesterol/BW (exceeding ±6%), −12.8% for GGT/RO, and 16.3% for GGT/BG (exceeding ±10%).

### 3.3 Longitudinal evaluation

A further mixed-effects model analysis of longitudinal EQA data from 08/2023 to 10/2024 provided mean ln bias estimates for individual EQAM batches (panel C of Figures 1–6), allowing assessment of between-batch variability for each sample type.

For cholesterol and urea, the variability in mean ln bias were markedly higher for the processed than for the native EQAM batches across almost all MPs (panel C of Figures 1, 2; Table 1A). The mean ln bias was within the range of ±6% for most of the processed batches, with the exception of the BW cohort in general and batch B5 (M_B_) for all collectives in the cholesterol measurements. The native EQAM batches, on the other hand, consistently exhibit a very constant mean ln bias for both analytes. For cholesterol, apart from batch B5 (M_B_), variations were more pronounced for the BE, OL and BW collectives, accompanied by a shift in the bias compared to those of the native EQAM batches. The corresponding calculated mean differences in ln bias between the sample types were 6.1% and 6.3% for the BE and OL collectives, and 14.9% for the BW collective (panel D of Figures 1–6). For urea, the observed variations in the mean ln bias of processed batches tend to be associated with a shift relative to the bias of the native batches, particularly for the BE, OL, SI and BG collectives. Whereas, all MP-specific mean differences in ln bias between the sample types were below 4.6% (panel D of Figures 1–6). For the measuremt of creatinine, considering enzymatic and Jaffé kinetic methods, variability in mean ln bias was largely similar for native and processed EQAMs (panel C of Figures 3, 4; Table 1), with only slight difference in bias between the two sample types (panel D of Figures 3, 4). The BE and OL collectives were notable exceptions. Apart from these exceptions, the mean ln bias for most batches using enzymatic methods (approximately ±6%) was lower than for Jaffé kinetic methods (approximately −4% to 15%) (panel C of Figures 3, 4; Table 1). In kinetic analyses, substantial scatter in mean ln bias was also observed for native EQAMs in some collectives. The MP-dependent mean differences in ln bias scattered between −7.6% and 5.2% for the enzymatic methods and between −3.3% and 12.1% for the use of Jaffé kinetic methods and (panel D of Figures 3, 4).

For GGT, mean ln bias for most processed EQAM batches ranged from approximately −6% to 11% across all collectives (Figures 5C, Table 1B). In contrast, native batches showed greater variability, with MP-dependent mean ln bias of up to ±16% for most batches. Consistent with the more frequently observed negative bias in the native samples, there was a shift compared to the processed batches for most MPs. Accordingly, the calculated mean differences in ln bias ranged from −4.3% to −11.3%, depending on the MP collective (Figure 5D).

For sodium analysis, mean ln bias ranged from −2.9% to 0.9% for processed batches across collectives, and from −1.3% to 0.5% for native EQAMs (Figure 6C; Table 1B). For all MP-specific bias, variability was pronounced for the processed EQAMs compared to the native EQAMs. Besides that, the BE and OL collectives exhibited a clear shift in the mean ln bias between both sample types (Figure 6C). The mean differnces in ln bias were between 0.03% and 1.65% depending on the MP collective (Figure 6D).

The longitudinal EQA data analysis showed rather low variabilities in MP-specific median ln bias for repeatedly distributed processed EQAMs between 08/2023 and 10/2024 (Supplementary Table S2). Analyte/MP-specific differences between the maximum and minimum median bias were <4% for most processed EQAM batches; larger differences were observed particularly for the SI and BG collectives and for creatinine (kinetic) and GGT.

## 4 Discussion

Building on the previously described approach of comparative EQA surveys using native human sera alongside the routinely distributed processed EQAMs (Vierbaum et al., 2026), the present study demonstrates that incorporating longitudinal data from processed EQAM batches enables a more comprehensive assessment of the respective contributions of MP-specific trueness and commutability to the observed total bias.

To minimize concentration-dependent effects, a prerequisite for meaningful comparison of bias between the two sample types is the selection of native materials with comparable concentration ranges to processed EQAMs. In this study, potential donors were pre-characterized for the target analytes approximately 2 months prior to sample collection. Documented analyte profiles supported candidate selection, and pre-characterization results were confirmed for most analytes despite the time interval and inherent intra-individual variability. However, previously observed low concentrations of cholesterol and urea could not be confirmed in the final native EQAMs.

Ultimately, compared with the native EQAMs, the processed EQAMs used in the 05/2024 survey showed considerably higher values for urea (103 mg/dL) and creatinine (5.24 mg/dL), but only slightly higher values for cholesterol (237 mg/dL) and sodium (148 mmol/L). A considerably lower value in the processed EQAMs compared with the native EQAMs was given for sodium (128 mmol/L), and a slightly lower value for cholesterol (108 mg/dL). However, the longitudinal analyses indicated that the observed bias patterns were not substantially affected by analyte concentration.

An important consideration when interpreting comparative EQA studies is the limited number of native EQAMs that can be included. Thus, the predefined commutability criteria should be interpreted as a pragmatic initial classification, as mean bias estimates derived from a limited number of native EQAMs and may be affected by sample-specific variability (panel B of Figures 1–6). Accordingly, analyte/MP pairings for which processed EQAMs were classified as potentially critical based on the criteria require further evaluation of bias variability across native EQAMs and processed EQAM batches. Potential variability in MP-specific bias among native EQAMs is of particular relevance, as it may indicate limited measurement reliability (Vierbaum et al., 2026). Ideally, such effects can be identified even with a small number of native materials, although the robustness of the assessment improves with a larger set of commutable materials.

Longitudinal analyses of processed EQAMs reveal whether observed bias patterns are reproducible across batches or attributable to individual batch-specific effects (panel C of Figures 1–6). As demonstrated by several analyte/MP combinations in this study, this complementary level of evaluation substantially improve interpretation of comparative EQA results and facilitate differentiation between MP-related susceptibility to interferences and batch-dependent material effects. Moreover, consistent batch-specific bias across surveys suggests that no relevant modifications were introduced by the MP manufacturers that could have influenced longitudinal bias analysis (Supplementary Table S2). However, it must be considered that small collective sizes, differences in participant composition or MP susceptibility to interfering factors may also affect the stability of the bias.

For cholesterol, urea, and enzymatic creatinine measurements, native EQAMs showed generally low (within ±4%) and consistent mean bias for most analyte/MP pairings, likely reflecting low endogenous interferences and a high degree of analytical comparability among MPs (panel A of Figures 1–3). Nevertheless, some trueness bias was identified for cholesterol using the BE, OL, and BW reagents, for urea using the RO, SI, and BG reagents, and for creatinine (enzymatic) using the AB, RO, and BG reagents.

In contrast, MP-specific bias was less consistent for processed EQAMs. While the comparative EQA survey for cholesterol and urea already indicated differences in bias between the two processed batches for some MPs, the longitudinal analysis revealed high variability in bias across multiple processed EQAM batches (panel C of Figures 1–3). Thus, longitudinal data provided a more comprehensive understanding of MP-specific commutability-related observations and demonstrated that such effects are highly batch-dependent, emphasizing the importance of evaluating individual batches rather than generalizing findings across processed materials. For cholesterol, one batch showed markedly pronounced negative bias across all MPs (except for RO), while all native EQAMs showed slight positive bias (panel C of Figures 1–3). This suggests either a batch-specific interference, or a higher concentration of an interfering substance. This is consistent with the known susceptibility of some enzymatic assays to matrix-related effects. In processed control materials, stabilization procedures, additives, or alterations associated with reconstitution may introduce MP-dependent bias relative to native patient samples, e.g., ammonia interference in urea assays resulting from ammonium sulfate precipitation (Bisswanger, 2014; Thomas, 2025).

In contrast, for creatinine (enzymatic), MP-specific bias was primarily observed to be consistent for both sample types. Accordingly, sample type-specific results of the comparative EQA surveys can be considered largely representative. As exceptions, clear indications of commutability issues were observed for three processed batches when using the BE and OL reagents. These commutability issues were confined to the three processed batches from control material manufacturer M_B_, suggesting that a specific additive in these materials may have caused markedly elevated creatinine results relative to the RMVs. Because the comparative survey included only processed EQAMs from manufacturer M_A_, this effect could only be identified through the longitudinal analysis. The study data did not allow any material manufacturer-specific trends to be identified for cholesterol or urea.

Bias patterns observed for Jaffé kinetic creatinine assay differed markedly from those observed for the enzymatic assay. Striking bias was observed for batch B1 (M_A_), hence, only the EQA results for batch B2 (M_A_) from the comparative EQA study were considered meaningful for inclusion in comparative analysis. Thus, the longitudinal analysis became particularly important to assess MP-specific bias patterns across multiple EQAM batches (Figure 4C).

High variability in bias was observed not only for processed but also for native EQAMs. This suggests that method-related limitations, rather than matrix effects alone, contribute to the observed variation and are consistent with the known limited analytical selectivity of the Jaffé reaction, which is susceptible to interference from non-creatinine chromogens (Dimeski and Kruger, 2021; Thomas, 2025). Furthermore, previously published trueness-based EQA programs for creatinine, employing commutable materials to assess the status of method standardization, demonstrated that Jaffé methods remain susceptible to method-dependent bias due to the limited analytical selectivity of the reaction, resulting in greater between-method variability compared with enzymatic creatinine assays (González-Lao et al., 2017; Ricós et al., 2019).

Considering the observed variabilities, most MP collectives showed only a slight and not critical shift in the average bias between all native and all processed batches. As exceptions, the BE and OL collectives exhibited clear shifts toward negative bias in three processed EQAM batches, all provided by control material manufacturer M_B_. These findings provide indications of commutability-related effects that were primarily attributable to specific batches or manufacturers, rather than reflecting an inherent limitation of processed control materials.

For GGT, native EQAMs often exhibited greater MP-specific bias than processed EQAMs (Figure 5A). Except for the BW collective, mean differences in bias between the sample types were either close to the lower acceptance limit for commutability or even exceeded it, as shown for the RO and BG collectives (Figure 5B). As enzyme activity measurements are sensitive to reaction conditions, slight variations in temperature or pH may affect reaction kinetics and introduce MP-dependent bias. However, such effects would be expected to occur largely independent of sample type. Longitudinal analysis of processed EQAMs showed generally low and consistent bias across most collectives (Figure 5C), indicating that GGT measurements were largely unaffected by lyophilization and additives. In contrast, variability in bias among native EQAMs was generally pronounced.

For native EQAMs, a correlation between the pronounced negative bias and low GGT activities (<20 U/L for batches N1 and N4, and <50 U/L for batch N3) was observed, suggesting a sensitivity-related limitation. GGT activity was measured by EQA participants using the IFCC primary RMP, which was also applied for target value assignment (Schumann et al., 2002; Thomas, 2025). As enzyme activity measurements rely on precisely controlled reaction conditions, even minor variations may influence reaction kinetics, particularly at low activity levels. Schumann et al. pointed out that achieving the required measurement temperature at the start of the reaction requires adequate pre-tempering of both sample and substrate solution due to the relatively large reaction volumes (Schumann et al., 2002). However, it should be noted that three processed EQAMs, B1 (M_A_), B3 (M_B_), B6 (M_C_), also had assigned RMVs <50 U/L but did not exhibit this trend toward pronounced negative bias. It is conceivable that the lack of stabilization in the native EQAMs led to a decrease in catalytically active GGT during shipment to the participating laboratories, although this was not confirmed in the parallel stability testing. Combined with the initially low GGT activity levels, even minor decreases in enzyme activity may result in substantial bias, depending on MP sensitivity. In this context, stabilized matrices may offer improved enzyme stability compared with native serum.

In this study, the comparative EQA approach turned out not to be suitable for assessing MP trueness and the commutability of processed EQAMs for GGT activities in the clinical reference interval (Thomas, 2025). Further EQA studies using additional native EQAMs exclusively at elevated GGT levels would be valuable for further assessment. Although the inclusion of such samples was intended in the present study, suitable donors with elevated GGT levels could not be identified during pre-selection.

For sodium, the comparative EQA study revealed slight negative bias for both native and processed EQAMs, which was marginally greater in the processed materials (Figure 6A). Across all MPs, however, the observed bias differences between the two sample types remained below 1.5% (Figure 6B), indicating only minor matrix-related effects based on the samples included. This tendency toward more pronounced bias in processed materials was confirmed by the longitudinal batch-specific EQA data, with most MP collectives showing higher variation in bias for processed than for native EQAMs (Figure 6C). The quite consistent bias observed for native EQAMs likely indicated minor non-selectivity, with slight trueness bias for the AB, BE, RO and BG collectives. This might be due to differences in selectivity or calibration of sodium ion-selective electrode (ISE) membranes. The BE and BW collectives showed a bias that was likely due to non-commutability for one processed EQAM batch (B4 (M_B_), or B1 (M_A_)). The BE, OL and BW collectives exhibited pronounced bias approaching the commutability limit for several of the processed EQAMs. Observed bias patterns need to be confirmed through additional data. However, lower sodium concentrations compared with the RMVs may occur with indirect ISE methods due to changes in the sample matrix, such as increased protein or lipid content affecting the measured sodium concentration. This systematic negative bias is particularly relevant for synthetic matrices (e.g., albumin-based solutions) or matrix alterations resulting from incomplete reconstitution of lyophilized EQAMs (Burnett et al., 2000; Thomas, 2025).

In summary, for cholesterol, urea, creatinine (enzymatic), and sodium, native EQAMs showed only minor bias for individual analyte/MP pairings, and generally consistent performance across MPs, indicating high analytical comparability. Slight variations in bias for individual MPs suggest a minor susceptibility to native matrices. In contrast, kinetic creatinine measurements showed more pronounced bias for some MPs and significant variability in MP-specific bias across native EQAMs, which is in line with the known susceptibility of the method to interferences. Pronounced bias for GGT was observed across all MPs, but only for native EQAMs with activities <50 U/L, suggesting potential stability-related limitations and highlighting the need for further evaluation using native samples with higher GGT activities.

Across the six analytes, cholesterol and urea showed the most pronounced indications of commutability-related effects, with multiple processed EQAMs exhibiting deviations from native sample behavior across several MPs. In contrast, such observations for creatinine and sodium were mainly restricted to specific analyte/MP pairings or individual batches. The reagent manufacturers BE and OL showed susceptibility to matrix-related effects across several analytes, indicating limited robustness against interferences introduced by processed control materials. Furthermore, some effects were clearly material-manufacturer dependent, e.g., all three EQAM batches from manufacturer M_B_ showed pronounced bias for the creatinine/BE and creatinine/OL pairings. Enzymatic creatinine measurements showed strongly positive bias, whereas Jaffé kinetic measurements showed strongly negative bias. Considering the limited number of samples, the observed bias patterns and related interpretations require further confirmation through additional data generated in EQA schemes or conventional commutability assessments.

Given the highly synthetic nature of processed control materials used in clinical chemistry and the large number of different MPs represented in EQA programs, complete commutability across all analyte/MP pairings cannot generally be expected. Nevertheless, knowledge of the commutability characteristics of individual EQAMs is essential for the reliable interpretation of EQA results and for drawing valid conclusions regarding the current status of measurement standardization.

Ultimately, these analyses demonstrate that EQA data from comparative surveys using native and processed materials provide valuable insights into MP trueness and thus the current status of standardization. Findings should be interpreted with caution, considering the aforementioned limitations, including the limited number of native samples and potential time-dependent changes during the longitudinal assessment. Differences in bias between sample types derived from a single comparative study should be interpreted carefully in terms of commutability, as conclusions may be affected by batch-specific bias. The conclusion of the findings would benefit from further comparative EQA surveys including native samples.

Importantly, the longitudinal analysis of processed EQAMs enabled a more reliable differentiation between reproducible MP-specific bias patterns and isolated batch-specific material effects, thereby substantially strengthening the interpretation of this comparative EQA results. It was found that commutability characteristics cannot be generalized across materials from the same manufacturer, as individual control materials may exhibit distinct commutability behavior.

To obtain such insights and complement ongoing bias monitoring of processed EQAMs, it is recommended that EQA providers should consider conducting periodic surveys using commutable materials (González-Lao et al., 2017; Jeong et al., 2021; Jones et al., 2022; Miller et al., 2008; Ricós et al., 2019; Vierbaum et al., 2024a). Trueness bias provides valuable feedback for MP manufacturers, whereas commutability-related bias across market-relevant MPs provides important information for control material manufacturers.

## Data Availability

The original contributions presented in the study are included in the article/Supplementary Material, further inquiries can be directed to the corresponding author.
